# Prognostic Role and Therapeutic Implications of Intravascular Optical Coherence Tomography Detected Coronary Plaque Microstructures in Patients with Coronary Artery Disease

**DOI:** 10.3390/jcm14228132

**Published:** 2025-11-17

**Authors:** Michele Russo, Elena Bacigalupi, Francesco Radico, Luca Scorpiglione, Filippo Luca Gurgoglione, Alessandro Russo, Carlo Vigna, Mattia Galli, Stefano Benenati, Rocco Vergallo, Rocco Antonio Montone, Umberto Benedetto, Giampaolo Niccoli, Francesco Prati, Marco Zimarino

**Affiliations:** 1 Department of Cardiology, SS. Annunziata Hospital, ASL2 Abruzzo, Via dei Vestini, 66100 Chieti, Italy; elenabaci96@gmail.com (E.B.); francescoradico@hotmail.it (F.R.); lscorpi94@gmail.com (L.S.); m.zimarino@unich.it (M.Z.); 2 Department of Medicine, University of Parma, 43125 Parma, Italy; filippolucagurgoglione@gmail.com (F.L.G.); gniccoli73@hotmail.it (G.N.); 3 Cardiology Unit, Fondazione IRCCS Casa Sollievo della Sofferenza, 71013 San Giovanni Rotondo, Italy; alessandrorusso17@hotmail.it (A.R.); cvigna57@gmail.com (C.V.); 4 Department of Medical-Surgical Sciences and Biotechnologies, Sapienza University of Rome, 04100 Latina, Italy; dottormattiagalli@gmail.com; 5 Maria Cecilia Hospital, GVM Care & Research, 48033 Cotignola, Italy; 6 Cardiovascular Disease Unit, IRCCS Ospedale Policlinico San Martino, IRCCS Italian Cardiology Network, 16132 Genova, Italy; stefanobenenatimd@gmail.com; 7 Interventional Cardiology Unit, Cardiothoracic and Vascular Department (DICATOV), IRCCS Ospedale Policlinico San Martino, 16132 Genova, Italy; roccovergallo@gmail.com; 8 Department of Internal Medicine and Medical Specialties (DIMI), Università di Genova, 16132 Genova, Italy; 9 Department of Cardiovascular and Pulmonary Sciences, Catholic University of the Sacred Heart, 00168 Rome, Italy; rocco.montone@gmail.com; 10 Department of Cardiovascular Sciences, Fondazione Policlinico Universitario A. Gemelli IRCCS, 00168 Rome, Italy; 11 Department of Cardiac Surgery, University “G. d’Annunzio”, 66100 Chieti, Italy; umberto.benedetto@asl2abruzzo.it; 12 Department of Cardiovascular Sciences, San Giovanni Addolorata Hospital, 00184 Rome, Italy; fprati61@gmail.com; 13 Centro per la Lotta Contro L’Infarto—CLI Foundation, 00182 Rome, Italy; 14 Department of Neuroscience, Imaging and Clinical Sciences, “Gabriele d’Annunzio” University of Chieti-Pescara, 66100 Chieti, Italy

**Keywords:** plaque microstructures, atherosclerosis, coronary artery disease, vulnerable plaque, prognosis, optical coherence tomography

## Abstract

Intracoronary optical coherence tomography (OCT) is a highly accurate and sensitive imaging tool capable of providing high resolution visualization of atherosclerotic coronary plaque morphology and microstructures in vivo. OCT has proven to be useful in clinical practice, particularly in percutaneous coronary intervention (PCI) guidance, assessment of stent-related complications, and elucidation of the pathobiological cause of acute coronary syndrome. Notably, OCT allows for the detection of specific plaque features (i.e., thin cap fibroatheroma, lipid-rich plaque, macrophage infiltration, healed plaques, microvessels, etc.) that are known to carry prognostic significance in the context of coronary artery disease (CAD). These insights may offer valuable information about the patient’s overall atherosclerotic background, potentially supporting more personalized secondary prevention strategies, including lifestyle modification and targeted pharmacologic therapies. Recently, the role of preventive PCI in plaques with high-risk features has also been investigated with promising—though still preliminary—results. In this narrative review, we primarily aim to discuss studies evaluating the prognostic value of OCT-identified coronary plaque microstructures. We also assessed potential therapeutic implications in the management of patients with CAD.

## 1. Introduction

Ischemic heart disease (IHD) is the leading cause of admission to intensive cardiac care units and death worldwide, and its absolute prevalence is expected to increase in the future, mainly because of population aging. In particular, it has been forecasted that IHD will cause 186.5 million of all-age disability-adjusted life-years (DALYs) and about 10.6 million of all-age deaths in 2050 [1,2]. Coronary atherosclerosis is the main cause of coronary artery disease (CAD) and IHD, and the study of CAD pathophysiology and consequent therapeutic implications represents the main topic of current research on IHD.

Intracoronary optical coherence tomography (OCT) is a highly sensitive and accurate diagnostic tool performed during coronary angiography that is able to study coronary plaque walls and microstructures in vivo [3]. OCT uses near-infrared light (wavelength of about 1.3 μm) to produce high resolution images (axial resolution is between 10 and 15 μm) generated by assessing the time delay of the light reflected or backscattered from the tissues (the vessel wall in the case of intravascular OCT) and from a predefined distance as a reference with a technique known as interferometry. The different properties of attenuation of the light by the tissues (i.e., lipid, calcium) are responsible for their diverse appearance in OCT images and define OCT penetration depth at these sites [3].

OCT, owing to its superior spatial resolution, has shown reliable diagnostic performance in elucidating the underlying pathobiological mechanisms of acute coronary syndrome (ACS), in assessing the causes of stent-related complications, and in guiding percutaneous coronary intervention (PCI) [4,5,6,7]. Notably, plaque microstructures imaged during OCT runs (i.e., thin-cap fibroatheroma—TCFA, lipid-rich plaques, healed plaques, microchannels, macrophage infiltration, etc.) have a documented prognostic relevance and are specific targets of preventive measures [8]. The beneficial role of preventive PCI has also shown promising results in the pioneering PREVENT study [9].

In recent years, numerous studies have investigated the prognostic impact of OCT-detected plaque features. Nevertheless, despite several reviews addressing the vulnerability characteristics of coronary plaque microstructures, to date no comprehensive review has summarized the existing evidence on their prognostic significance. Hereby, in this narrative review, we discuss the available data relative to the clinical significance of OCT-detected plaque microstructures in native coronary arteries. We also discuss possible therapeutic strategies for plaque stabilization and reduction of plaque-related future cardiovascular (CV) events.

## 2. Prognostic Significance of Plaque Microstructures Identified by OCT Imaging

An inclusive search of electronic databases, including PubMed, EMBASE, Web of Science, and Google Scholar, was performed to identify studies published from January 2010 to September 2025 assessing the relationship between OCT-detected plaque microstructures and clinical events at follow-up. References listed in the selected articles were also investigated. The following searched terms or phrases were used: “thin cap fibroatheroma and optical coherence tomography and events”, “thin cap fibroatheroma and optical coherence tomography and prognosis”; “lipid and optical coherence tomography and events”, “lipid and optical coherence tomography and prognosis”; “healed coronary plaque or layered coronary plaque and optical coherence tomography and events”, “healed coronary plaque or layered coronary plaque and optical coherence tomography and prognosis”; “macrophage and optical coherence tomography and events”, “macrophage and optical coherence tomography and prognosis”; “cholesterol crystal and optical coherence tomography and events”, “cholesterol crystal and optical coherence tomography and prognosis”; “microvessel or microchannel or intimal vasculature and optical coherence tomography and events”, “microvessel or microchannel or intimal vasculature and optical coherence tomography and prognosis”; “calcification or calcium or calcified nodule and optical coherence tomography and events”, “calcification or calcium or calcified nodule and optical coherence tomography and prognosis”; “plaque rupture and optical coherence tomography and events”, “plaque rupture and optical coherence tomography and prognosis”. Studies were considered eligible if (1) they included patients undergoing coronary angiography and concomitant intracoronary OCT imaging; (2) reported CV events at clinical follow-up; and (3) described an association between at least one coronary plaque microstructure as assessed by OCT and future CV events.

**Figure 1:**
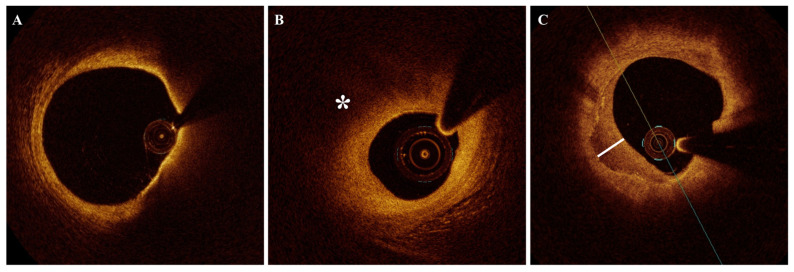
OCT imaging of TCFA, lipid-rich plaque, and healed plaque. (**A**) TCFA; (**B**) lipid-rich plaque (asterisk indicates lipid pool); (**C**) healed plaque (line indicates the layer of tissue). OCT = optical coherence tomography; TCFA = thin cap fibroatheroma.

**Figure 2:**
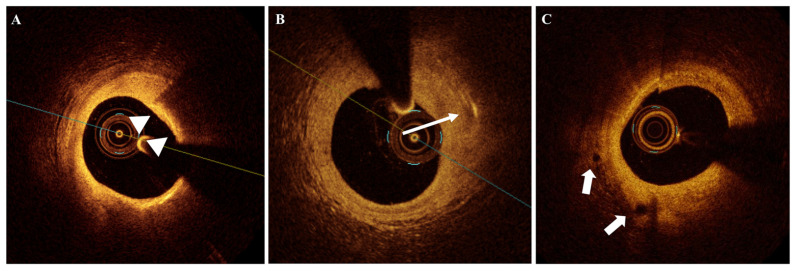
OCT imaging of MØI, CCs, and microvessels. (**A**) MØI (indicated by arrowheads); (**B**) presence of CCs (indicated by arrow); (**C**) microvessels (indicated by arrows). CCs = cholesterol crystals; MØI = macrophage infiltration; OCT = optical coherence tomography.

**Figure 3:**
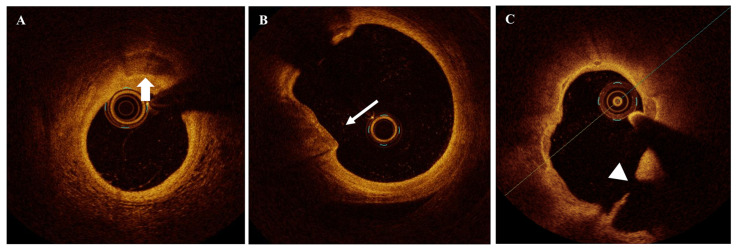
OCT imaging of spotty calcification, calcified nodule, and previous rupture. (**A**) spotty calcification (indicated by arrow); (**B**) CN (indicated by arrow); (**C**) previous plaque rupture (cavity is indicated by arrowheads). CN = calcified nodules; OCT = optical coherence tomography.

Figure 1, Figure 2 and Figure 3 show the OCT appearance of different examined plaque microstructures. Table S1 summarizes the studies assessing the prognostic impact of coronary plaque features evaluated by intracoronary OCT imaging.

### 2.1. TCFA

According to histological studies, TCFA is considered the prototype of vulnerable plaques and the precursor of plaque rupture [10]. In histology samples, it is usually characterized by large necrotic cores covered by a thin layer of fibrous tissue (representing the fibrous cap), high macrophage and lymphocyte intimal infiltration, and deficient smooth muscle cells (SMCs) [11,12]. In a post-mortem study, Burke et al. [13] found that 95% of patients who died suddenly from coronary thrombosis due to plaque rupture had a fibrous cap < 65 μm. The proximal sites of major epicardial arteries, in particular the left descending artery (LAD), are the most frequent sites of TCFA localization in pathology and intravascular imaging studies [10,14]. OCT imaging showed a high ability to identify TCFA, defined as a lipid plaque having a lipid arc in circumference >90° enclosed by a fibrous cap under a definite threshold (Figure 1A). A minimal cut-off of 65 μm is usually adopted to define a fibrous cap as thin at OCT imaging, although different values have been used by other studies [3]. OCT studies reported that TCFAs have been related to plaque rupture and have been associated with other “vulnerable” plaque features such as macrophage infiltration (MØI), microvessels, and cholesterol crystals [15,16,17,18]. TCFA has also been associated with higher pericoronary adipose tissue attenuation (PCAT) [19] and higher levels of blood markers of systemic inflammation [20], thus suggesting a relationship between TCFA and inflammation.

TCFA as assessed by OCT imaging has been strongly associated with future CV events [8,21,22,23,24,25,26,27,28]. In a recent OCT-imaged three-epicardial coronary vessel study including 1312 ACS patients with five-year follow-up, Dai et al. [21] reported that TCFA (defined as lipid plaque with maximum lipid arc > 180° and thinnest fibrous cap thickness (FCT) < 65 μm) in non-culprit lesions (NCL) was associated with increased risk of patient-level NCL-related major adverse CV events (MACEs) (hazard ratio [HR] 2.50 [95% CI: 1.48–4.20]), enclosing a composite of cardiac death, non-fatal myocardial infarction (MI), and unplanned coronary revascularization. Biccirè et al. [22], in the long term follow-up of the Relationship Between OCT Coronary Plaque Morphology and Clinical Outcome (CLIMA) [8] study, including 1003 patients and evaluating the prognostic significance of OCT plaque features in untreated proximal LAD, reported that TCFA with FCT < 75 μm was strongly associated with the composite hard endpoint of cardiac death and target-segment MI (HR 3.46 [95% CI: 2.17–5.53]). Interestingly, in the COMBINE OCT-FFR, merging both hemodynamic interrogation by fractional flow reserve (FFR) and imaging assessment by OCT of coronary plaques in patients with diabetes mellitus, the presence of TCFA (defined as lipid-rich plaque with lipid arc > 90° and FCT ≤ 65 μm) in FFR-negative lesions was an independent predictor of future events (HR 2.89 [95% CI: 1.61–5.20]) in a follow-up time up to 5 years [23]. Notably, a meta-analysis by Gallone et al. [24] confirmed these observations, showing that TCFA was associated with MACEs both at the patient-level and lesion-level. Finally, Yamaji et al. [27] reported that TCFA (defined as lipid plaque with minimum FCT ≤ 65 μm) was significantly associated with a more than 2-fold higher three-year risk of ischemia-driven non-target vessel revascularization, primarily related to revascularization in the imaged region. Accordingly, it is important to emphasize that not all TCFAs that have undergone destabilization cause ACS. Plaque healing may occur, and the generation of a new layer of tissue may favor plaque growth and negative vessel remodeling [29,30]. In this regard, serial OCT studies reported that TCFA was a strong predictor of plaque progression [31,32], and was associated with the presence of healed plaques [33].

### 2.2. Lipid-Rich Plaque and Lipid Burden

Lipids are identified by OCT imaging as signal-poor regions with diffuse borders and high attenuation properties with an overlying signal-rich band with discrete margins representing the fibrous cap [3] (Figure 1B). Considering the high attenuation features, penetration depth at lipid sites is markedly reduced, impairing the ability of OCT to assess structures behind lipid pools.

Previous histopathological validation studies reported high agreement of lipid assessment between OCT and pathology specimens [34].

Lipid-rich plaques (LRP) in OCT imaging are usually defined as plaques with a circumferential lipid arc > 90°. Semiquantitative measures such as lipid arc, lipid length, and lipid index (mean lipid arc x lipid length) are usually used to calculate OCT-imaged lipid burden [3]. In a previous three-vessel OCT study in patients with CAD, a prevalence of 1.3 LRP per patient was reported, and patients with ACS showed higher lipid burden than those with stable angina [35]. LRP has been associated with plaque features such as MØI, microvessels, and healed plaques [17,33,36]. Moreover, LRP was found to be an independent predictor of rapid plaque and calcification progression [31,37].

Many OCT investigations assessed the prognostic impact of LRP and lipid burden, mainly showing a relationship between these features and future CV events [8,22,26,38,39,40]. Xing et al. [38], in a four-year follow-up study of patients undergoing PCI, reported that LRP in NCL of the target vessel was associated with increased non-culprit lesion MACE (HR 2.06 [95% CI: 1.05–4.04]), defined as a composite of cardiac death, acute MI, and ischemia-driven revascularization. In the study, a larger lipid burden in LRP was found in patients with non-culprit MACE. Kubo et al. [26], in a study including 1378 patients with a median follow-up period of 6 years, similarly reported that LRP at a non-culprit lesion was strongly significantly associated with future ACS (HR 12.67 [95% CI: 6.82–23.57]) attributed to a non-culprit plaque imaged by OCT at baseline. Importantly, in the previously cited CLIMA study and its five-year follow-up report, lipid plaques in the proximal LAD with lipid arc circumferential extension >180° had increased risk of future events (HR 2.4 [95% CI: 1.2–4.8]; HR 2.2 [95% CI: 1.4–3.5], respectively) [8,22]. Another study derived from the CLIMA assessing the use of automated software to calculate lipid burden reported that the automated maximum OCT-derived lipid core burden index (LCBI) in 4 mm predicted CV events and was associated with vulnerable plaque features [39]. Interestingly, in a report from the COMBINE OCT-FFR trial, Fabris et al. [40] suggested that, in 390 diabetic patients undergoing OCT of non-ischemic lesions, TCFA had a stronger association with CV events than overall LRP, showing that thick-cap fibroatheroma LRP had a similar risk of future events to non-LRP. Finally, many studies described the relationship between lipid plaque burden and worse PCI outcomes (mainly related to stent edge dissection, tissue protrusion, and incomplete apposition), reduced Thrombolysis in Myocardial Infarction (TIMI) flow post-PCI, and periprocedural biomarker elevation, the latter probably due to lipid debris distal embolization [34].

### 2.3. Healed Plaques

Healed coronary plaques are lesions presenting signs of previous plaque destabilization undergoing a healing process. Also called “layered plaques” for their appearance, they are defined at OCT imaging as plaques presenting one or more layers with different optical densities and a clear demarcation from underlying components [33] (Figure 1C). A recent pathology-OCT validation study reported high agreement between OCT imaging and histology in healed plaque identification, paving the way to the study of this plaque phenotype in vivo [41,42]. In histology studies, healed lesions have been found in the coronary arteries of >50% of patients dying suddenly from coronary death who had no evidence of previous MI and have been described at sites with greater luminal stenosis, suggesting their role in plaque growth and vessel luminal narrowing [43,44]. Accordingly, OCT studies reported that healed plaques were associated with higher stenosis severity and plaque vulnerability (more TCFA, lipid plaques, and macrophages) and were found to independently predict rapid plaque progression [29,31,33,45]. Stable angina pectoris (SAP), multivessel disease, complex lesion, and diameter stenosis >70% were found to be independent predictors of healed plaque at the culprit lesion in a previous study [46].

Several recent OCT studies have evaluated the clinical significance of healed plaques in the coronary arteries, demonstrating a strong association between healed lesions and the need for future PCI [8,47,48,49,50,51,52,53,54,55,56,57]. In the study by Kurihara et al. [47] including 265 patients with either ACS or SAP, those with healed coronary plaques had a higher future revascularization rate at two-year follow-up (odds ratio [OR] 3.096 [95% CI 1.148–8.356]) but similar “hard outcomes” of cardiac death and ACS than those without healed lesions. In another recent report by Yi et al. [48] studying 553 NCL in ACS patients followed up for 6 years, the authors found similar results, showing that patients with new layered lesions at follow-up had a higher risk of NCL-MACEs (HR 2.52 [95% CI 1.20–5.33]), mainly determined by non-culprit-plaque-related coronary revascularization. Other studies confirmed the association between healed plaques and increased risk of revascularization, showing similar outcomes about future ACS, cardiac death, or death from any cause [49,50,51]. Fracassi et al. reported increased prevalence of all-cause rehospitalization in patients with ACS with culprit healed lesions, while no differences in the rate of future death, ACS, or revascularization were found between the groups [52]. On the other hand, other reports with shorter follow-up found no differences in clinical outcomes between patients with and without healed plaques [53,54,55]. In the CLIMA study, having as a primary endpoint a composite of cardiac death and target segment MI, healed plaques were not associated with the main outcome [8]. Interestingly, in a prospective clinical follow-up study, patients with recurrent ACS had the lowest prevalence of healed lesions as opposed to those with single acute MI and those with long-standing SAP [56]. Accordingly, a dual significance of healed plaques in the coronary arteries has been suggested: although healed plaques are signs of plaques undergoing previous destabilization, patients with these features may have a more effective plaque healing process with greater protection against occlusive thrombosis and recurrent ACS. On the other hand, plaque healing, through thrombus organization and deposition of new layer of fibrous tissue, may contribute to plaque progression, negative vessel remodeling and ischemia, potentially requiring future revascularization [30,31,42]. Finally, regarding post-PCI outcomes, healed plaques have been associated with reduced stent expansion ratio and mean stent eccentricity index, potentially requiring more aggressive balloon dilatation to obtain optimal stent results [57].

### 2.4. Macrophage Infiltration (MØI)

Coronary plaque inflammation is thought to play a key role in the mechanisms of plaque growth and plaque rupture, with macrophages representing the major inflammatory cells involved in the process. In histology studies including patients who died from sudden coronary death, macrophages have been colocalized with ruptured plaques and with occlusive thrombi [10]. A previous study comparing OCT and pathology reported that OCT can identify sites of the fibrous cap with high macrophage density, appearing as punctate sites with high reflecting properties [58]. The use of normalized standard deviation (NSD) for quantifying MØI has been proposed, as it shows an excellent correlation with the percentage of CD68 staining in fibroatheroma; however, alternative approaches have also been described [58,59,60,61,62]. MØI are conventionally defined in OCT imaging as signal-rich, distinct, or confluent punctate regions that exceed the intensity of background speckle noise with a dark shadow behind aggregates [3] (Figure 2A). According to the validation study valuing macrophage density into the fibrous cap, MØI should be assessed only in fibroatheromas [3,58]. Macrophage density measured by OCT was found to be higher in unstable patients, and surface MØI was related to unstable clinical presentations [63]. In addition, macrophage density was higher in TCFA and was found to positively correlate with baseline white blood cell count and LRP and inversely relate to fibrous cap thickness [16]. High C-reactive protein was also independently associated with MØI within the culprit plaques in ACS patients in another study [64], and PCAT was found to be higher in patients with culprit plaque MØI [65].

Clinical studies have confirmed the relationship between MØI and adverse CV outcomes [8,22,66,67,68,69,70]. The previously referenced CLIMA study reported that at both one-year and five-year follow-up, OCT-identified MØI in the untreated proximal LAD was associated with increased risk of hard events (HR 2.7 [95% CI: 1.2–6.1]; HR: 1.81 [95% CI: 1.09–3.01], respectively) [8,22]. In a sub-study of the CLIMA, the prognostic role of macrophage burden was also assessed, and large macrophage arcs (>67°) and superficial macrophages (defined as a distance from intima-lumen contour to macrophage string < 0.12 mm) were found to be independent predictors of the main outcome [66]. In the OCT-FORMIDABLE study examining 209 patients with ACS undergoing OCT imaging, necrotic core with MØI in the culprit lesion was an independent predictor of the composite endpoint of death from cardiac causes, non-fatal MI, and clinically driven target vessel revascularization at a median follow-up of 12.6 months (HR 3.3 [95% CI: 1.6–6.6]) [67]. Colocalization of MØI and calcifications has also been related to future events [68]. In a prospective study, Fracassi et al. found that, among 156 ACS patients, MØI in the culprit plaque was an independent predictor of recurrent ACS (OR 3.145 [95% CI: 1.458–9.587]) at three-year follow-up [69]. Conversely, in another study of patients with stable CAD undergoing OCT imaging of the culprit vessel, MØI were not associated with future adverse CV outcomes [20]. Finally, Montone et al. reported that, in ACS patients with plaque erosion as the pathobiological mechanism of ACS, those with MØI at the culprit site had worse outcomes than those without MØI (HR 2.95 [95% CI: 1.09–8.02]), mainly driven by cardiac death and future revascularization in the target vessel and in the non-target lesions (median follow-up of 2.5 years) [70].

### 2.5. Cholesterol Crystals (CCs)

Cholesterol crystals (CCs) are the product of free cholesterol crystallization both in foam cells and in the extracellular space of atherosclerotic plaque intima derived from an imbalance in cholesterol homeostasis [71]. It has been demonstrated that CCs may trigger local inflammation by induction of the complement cascade and activation of the pyrin domain–containing 3 (NLRP3) inflammasome. In addition, pathology studies visualized sharp-tipped CCs perforating the fibrous cap and reported that CCs were associated with plaque disruption, plaque thrombosis, and plaque size, suggesting their potential role in plaque progression and destabilization [72,73].

OCT validation studies showed that the sensibility of OCT as compared to pathology to identify CCs was 26% and 68%, although its specificity was higher (100% and 92%, respectively) [74,75]. CCs are classically defined at OCT imaging as thin, linear regions of high intensity, usually in proximity to a lipid-rich plaque [3] (Figure 2B). The absence of back shadow behind the high-intensity regions helps to differentiate CCs from MØI. In OCT studies, CCs have been associated with coronary plaque rupture and other microstructures such as TCFA, MØI, microvessels, and calcifications [76,77,78].

The prognostic significance of CCs as assessed by OCT imaging in CAD patients has been reported by some studies, which showed heterogeneous results [8,22,79,80,81]. Fujiyoshi et al. [79] investigated the clinical impact of OCT-detected CCs at the culprit lesion in 340 CAD patients (both ACS and SAP) treated with PCI and followed up for 1 year. They found that the incidence of MACEs (defined as a composite of cardiac death, non-fatal MI, target and non-target vessel revascularization) was higher in patients with CCs at the culprit lesion (mainly driven by non-target vessel revascularization), although CCs did not independently predict MACEs when included in a model with other OCT features [79]. Similarly, in the CLIMA study, CCs were not associated with the composite outcome [8,22]. In a recent report from the OPTICO-ACS study program, Nelles et al. stated that among 346 ACS patients, those with CCs at the culprit lesion had an increased risk of MACE+ compared to those without CCs at two-year follow-up (HR 1.705 [95% CI: 1.025–2.838]) resulting mainly from higher rates of target vessel revascularization and higher rates of unstable or progressive angina [80]. Finally, Usui et al., studying 735 NCL in 566 patients with both ACS and SAP, found that patients with untreated NCL containing CCs and low-intensity areas without attenuation (hypothesized as representative of intraplaque hemorrhage) had an increased risk of NCL-MACEs (HR 3.09 [95% CI: 1.27–7.50], defined as a composite of cardiac death, MI, or ischemia-driven revascularization related to NCL, mainly caused by ischemia-driven revascularization at a mean follow-up of 2.5 years [81].

### 2.6. Microvessels

Plaque angiogenesis implies the formation of intimal neovessels, largely deriving from adventitial vasa vasorum, triggered by local plaque microenvironment. Newborn vessels contribute to “nourish” atherosclerotic lesions, favoring inflammatory cells, cytokines, and cholesterol entrance into the intima [82]. The rupture of the leaky walls of intimal microvessels may be responsible for intraplaque hemorrhage (IPH), causing abrupt plaque expansion, free cholesterol accumulation from erythrocyte membranes, increased oxidative stress, and local inflammation. Pathology studies reported that microvessel density was higher in ruptured plaques of human aorta, and it was increased in lesions with severe MØI, TCFAs, and at sites with IPH [83].

OCT imaging showed a discrete accuracy to identify microvessels (also defined as microchannels) as compared to pathology, described at OCT imaging as “signal-poor voids that are sharply delineated and can usually be followed in multiple contiguous frames” [3,84,85] (Figure 2C). An intravascular imaging study reported that microchannels were present in about 38% of lesions in CAD patients and were associated with intravascular ultrasound (IVUS)-assessed positive remodeling, OCT-detected TCFA, and elevated hs-CRP levels [86]. Microvessels were also associated with higher prevalence of intimal laceration, vulnerable plaque features, and increased risk of slow flow phenomenon after stent implantation [87]. In a study by Uemura et al., the presence of OCT-imaged microvessels at baseline in non-significant coronary plaques of 53 CAD patients predicted angiographic plaque progression after seventh-month follow-up [88]. Similar findings about plaque progression were reported by other studies [32,89,90].

Some studies have investigated the relationship between plaque microvessels and future clinical outcomes with inconsistent results [8,21,22,26,51,56,91]. Xu et al. [91] investigated the clinical impact of microvessels assessed by using intracoronary OCT in 535 patients with CAD either undergoing or not undergoing PCI. They found that, among patients undergoing PCI with stent implantation (340 patients), those with intraplaque microvessels had higher prevalence of periprocedural MI (4.0% vs. 1.6%, *p* < 0.005) and intraprocedural no-reflow (24.0% vs. 1.6%, *p* < 0.005) than those without. Conversely, patients not undergoing PCI (195 patients) were followed up for a median of 3.2 years, and those with intraplaque microvessels showed higher prevalence of clinically driven target lesion revascularization (12.4% vs. 1.4%, *p* < 0.001) at follow-up [91]. Other studies did not find an association between OCT-assessed microvessels and “hard outcomes” such as future ACS, MI, recurrent ACS, and cardiac death [8,21,22,26,56]. Similarly, in a recent post-hoc analysis of the COMBINE OCT-FFR trial, microvessels were not associated with the primary composite outcome at five-year follow-up [51].

### 2.7. Calcifications

Calcification is a key feature of the atherosclerotic process and reflects different phases of plaque biology, contributing to atherosclerosis progression and plaque destabilization [92].

OCT imaging showed very high accuracy for the identification of intimal calcification, described as signal-poor regions with sharply delineated borders and limited shadowing [3,93] (Figure 3A). According to calcium arc and calcium length, different dimensional subtypes of calcium deposits (microcalcification, spotty calcification, and large calcifications) and quantification of calcium burden have been assessed by OCT imaging [94,95]. Intracoronary OCT can recognize calcified nodules (CN) (Figure 3B) and other calcified plaque subtypes reported as possible pathobiological mechanisms of ACS at the culprit lesions [96].

The role of calcification in clinical outcomes remains a matter of ongoing debate. Pathological data have shown that patients who died from myocardial infarction had more frequent calcifications compared with individuals without a history of CV disease who died from non-cardiac causes. However, calcification was not associated with plaque instability, and the overall calcium burden showed an inverse relationship with MØI [97]. In vivo studies (coronary computed tomography and intravascular imaging studies) confirmed these data, reporting higher prevalence of obstructive CAD in patients with elevated coronary artery calcium scores (CACs), but reduced grade of local inflammation and vulnerability in the case of extensive calcifications and high calcification burden [94,95,98]. About calcification subtypes, spotty calcifications have been more frequently found in ACS patients than SAP and have been related to high-risk plaque features and signs of vascular inflammation [99,100,101]. Modification of tensile stress of the vessel wall by small calcium gatherings has also been suggested as a possible factor favoring plaque disruption [102]. Calcium burden was also found to negatively correlate with positive remodeling [103], and calcifications were associated with plaque progression [104].

In agreement with this evidence, a dual clinical connotation of calcification in the coronary arteries has been hypothesized: small calcium burdens and deposits would reflect more biologically “active” plaques with a higher degree of local inflammation and increased risk of progression, while extensive and bulky calcifications would indicate more “stable” plaques in advanced stages of the atherosclerotic process, with lower inflammatory infiltrates and minor lipid burdens but more extended atherosclerotic disease [95,105].

Calcium nodules were reported in about 4.2% of coronary lesions (both in stable and ACS patients) and were more frequently found in the ostial or mid-right coronary artery in a previous study [106]. They were the cause of coronary thrombosis in 2–7% of patients with sudden death and were associated with age, hemodialysis, diabetes, and severe calcifications [10,106,107].

Many studies assessed the prognostic impact of calcifications on future MACEs [21,26,38,56,108,109,110,111,112,113]. In the previously cited large population study of Dai et al. with a median follow-up of 4.1 years, calcifications in untreated non-culprit coronary lesions were predictors of future events (HR 1.93 [95% CI: 1.10–3.37]) at the lesion-level, although no specific data about calcium burden and subtypes were stated in the study [21]. Conversely, no statistically significant differences in non-culprit plaque MACEs and ACS were described in non-culprit lesions with calcifications in other studies [26,38].

Among calcium subtypes, spotty calcifications have been associated with future events [108,109]. Nelles et al. [108] reported that among 155 ACS patients undergoing PCI, those with spotty calcifications at the culprit lesion had worse outcomes (HR 3.29 [95% CI: 1.05–10.32]), defined as the composite of cardiac death, non-fatal MI, clinically driven target vessel revascularization, and re-hospitalization due to unstable or progressive angina at a median follow-up of 10.4 months. Similarly, Vergallo et al. [56] reported that spotty calcifications were more frequent in patients with recurrent ACS than in those with single acute MI and long-standing stable angina.

About CN in a sub-analysis of the CLIMA study, Prati et al. demonstrated that the presence of CN with disruption of the superficial intimal fibrous layer in untreated proximal LAD lesions was a strong independent predictor of future events (composite of cardiac death and target lesion MI) at one-year follow-up (HR 6.58 [95% CI: 2.7–15.8, *p* < 0.001]), whereas overall CN did not predict MACEs in the CLIMA study [8,109]. CN in patients in hemodialysis undergoing PCI were reported to have increased risk of MACEs (HR 4.93 [95% CI: 2.07–11.76]) driven by either all-cause and CV death or target-lesion revascularization at one-year follow-up [110]. Other studies suggested the positive association between CN in different clinical settings (culprit lesion in ACS patients, CN in patients with end-stage renal disease in hemodialysis, and newly developed CN in untreated calcified lesions) and adverse future CV outcomes [111,112,113].

The relationship between calcifications and CN and negative post-PCI outcomes is well known with either the use of drug-eluting stent, often requiring adequate lesion preparation with specific calcium-modifying devices to reduce the risk of stent underexpansion, or drug-coated balloons, the latter being associated with poor outcomes in calcified lesions and CN, mostly related to increased risk of target lesion revascularization [114,115,116,117,118].

### 2.8. Previous Ruptures (Cavities Within the Plaque in Stable Patients)

Plaque rupture (PR) is the main pathobiological cause of coronary thrombosis and sudden coronary death in pathology studies [10] and the cause of ACS related to plaque destabilization in intravascular imaging reports [119]. As previously indicated, PR can be recognized at OCT imaging by fibrous cap discontinuity over a lipid-rich necrotic core, typically associated with an intraplaque cavity and superimposed thrombus. Cavities without thrombi can also be identified at the sites of previous plaque rupture or in acute ruptures treated with antithrombotic or thrombolytic therapies [3] (Figure 3C).

Pathology evidence reported that PRs can be found in patients who died for non-cardiac causes without evidence of acute MI [120]. Similarly, three-coronary vessel OCT studies reported non-infarct-related non-culprit PR in about 20% of patients with CAD [104,121]. Plaques with previous rupture at the non-culprit sites were more frequently found in ACS than stable patients, and non-culprit plaques with cavities showed higher prevalence of TCFA, microvessels, MØI, CCs, calcification, spotty calcifications, and thrombus at the lesion-level than non-culprit lesions without PR [121].

Clinical significance of PR unrelated to acute MI at non-culprit sites has been investigated in few OCT studies [38,121,122]. In a recent OCT patient-data pooled analysis from COMBINE OCT-FFR and PECTUS-obs studies including a total of 810 patients with either acute or chronic coronary artery disease, untreated FFR-negative non-culprit lesions showing plaque rupture had more than 2-fold higher risk of native MACEs (composite of all-cause mortality, nonfatal MI, or unplanned revascularization excluding stent-failure-related events and nonattributable events) and more than 3-fold higher risk of target lesion failure (composite of cardiac death, target vessel MI, or target lesion revascularization) than non-culprit lesions without PR over a median follow-up of about 2 years [122]. Another study showed that patients with non-culprit PR assessed by OCT imaging, of whom only 13% underwent PCI at index OCT, reported higher rates of non-target lesion revascularization (11.8% versus 4.4%; *p* = 0.039) at one-year follow-up, of which two-thirds occurred at sites of non-culprit PR [121]. Conversely, in the study by Xing et al., no differences in non-culprit lesion MACEs were found between non-culprit lesions with and without PR undergoing PCI [38]. Upcoming studies will clarify the significance of previous rupture in the coronary arteries for future events.

## 3. Therapeutic Implications

Many drugs have shown effects on plaque features as shown by various imaging modalities (OCT, IVUS, coronary computed tomography angiography—CCTA, magnetic resonance imaging, etc.). In the present section, we focused on pharmacological agents with the strongest evidence of plaque structure modification assessed by OCT imaging. We also discuss the potential role of preventive PCI in vulnerable plaques.

### 3.1. Lipid-Lowering Therapies

Lipid-lowering therapies are the cornerstone of CV prevention and play a central role in reducing LDL cholesterol (LDL-C) levels. Recent evidence showed that these drugs may modify the composition and stability of atherosclerotic plaques. In particular, achieving very low LDL-C levels has been associated with smaller lipid arc, and thicker fibrous caps, and an inverse correlation between LDL-C and fibrous cap thickness has been described [123]. A meta-analysis of 12 randomized control trials also reported a strict correlation between lipid-lowering therapies and thicker fibrous cap and reduced maximum lipid arc [124]. Statins have been the first-line agents since the early 1990s, with consistent evidence from large randomized trials and meta-analyses demonstrating a strong, dose-dependent relationship between LDL-C lowering and reduction in CV events [125]. In addition, statins have been shown to reduce atherosclerotic plaque progression and favor plaque regression by significantly reducing both total atheroma volume (TAV) and percent atheroma volume (PAV) [126,127,128,129,130,131,132,133,134]. Numerous studies reported that statins act on plaque composition by increasing fibrous and calcified components, by reducing lipid content and necrotic core, and by increasing fibrous cap thickness [135,136,137]. The YELLOW trial demonstrated a marked reduction in lipid core burden in patients treated with high-dose rosuvastatin [135], while OCT-based studies such as EASY-FIT confirmed increases in fibrous cap thickness and decreases in the grade of OCT-derived macrophages with statin therapy [136]. CCTA analyses, such as in the PARADIGM study, also observed an increase in calcified plaque and a reduction in high-risk features such as positive remodeling [137,138].

Ezetimibe, when added to statin therapy, provides an additional LDL-C reduction and clinical benefit, as demonstrated in the IMPROVE-IT trial [139]. Meta-analyses have further shown that combination therapy with statins and ezetimibe results in greater plaque volume and TAV reduction compared to statin monotherapy [140,141]. However, the evidence on the impact of ezetimibe on plaque composition is mixed. Some imaging studies, such as those by Hibi et al., found no significant differences in PAV or key plaque features (fibrous, calcified, or lipid content) when ezetimibe was added to statin therapy [142]. Nevertheless, other studies have suggested modest improvements: for instance, Habara et al. reported a significant increase in fibrous cap thickness with fluvastatin-ezetimibe combination therapy versus fluvastatin alone [143]. An OCTIVUS sub-study reported lower prevalence of CCs in patients treated with ezetimibe than placebo [144].

PCSK9 inhibitors, including monoclonal antibodies such as evolocumab and alirocumab, have revolutionized lipid-lowering therapy by enabling profound reductions in LDL-C levels, with a corresponding significant decrease in MACE [145,146]. These agents have also been investigated for their effects on plaque morphology. The GLAGOV trial demonstrated greater reductions in TAV among patients treated with evolocumab compared to statin therapy alone [147], while the ODYSSEY J-IVUS trial, using alirocumab, showed a non-significant trend towards plaque regression [148]. Recent imaging studies using OCT have highlighted the ability of PCSK9 inhibitors to promote plaque stabilization. The HUYGENS trial found that evolocumab significantly increased fibrous cap thickness, reduced maximum lipid arc, and decreased macrophage index throughout arterial segments and LRP regions [149]. Similarly, the PACMAN-AMI trial provided compelling evidence of plaque regression and compositional change in non-culprit coronary arteries of post-acute MI patients treated with alirocumab in addition to high-intensity statins. Compared to statin therapy alone, combination therapy resulted in greater reductions in PAV, lipid burden, and macrophage content, along with a greater increase in fibrous cap thickness [150].

Finally, inclisiran, a small interfering RNA (siRNA) targeting hepatic PCSK9 synthesis, achieves a ~50% LDL-C reduction with twice-yearly subcutaneous administration [151]. Although outcome data from the ORION-4 trial are awaited, there is currently limited evidence on inclisiran effects on plaque composition. The ongoing VICTORION-PLAQUE study is expected to provide further insights into its potential role in plaque stabilization [152].

### 3.2. Colchicine

Increasing evidence showed that anti-inflammatory therapy represents a new target to fight atherosclerotic disease [153,154]. Colchicine is an anti-inflammatory medication that acts by inhibiting microtubule formation and the polymerization of tubulin, resulting in suppression of the inflammatory response [155]. The LoDoCo2 trial [156] revealed that colchicine reduced the risk of primary composite CV events by 31%, thought to be attributable to its anti-inflammatory effects. In addition, in a recent meta-analysis including 12,869 patients, colchicine therapy was associated with a reduced risk of MI, coronary revascularization, stroke, and hospitalization for CV causes compared to placebo, although no differences emerged for CV and all-cause deaths [157].

Colchicine has shown positive results on modifying plaque composition [158,159]. The COLOCT trial including 128 patients with CAD, reported that patients treated with colchicine had significantly higher minimal fibrous cap thickness, reduced average lipid arc and mean angular extension of macrophages at OCT imaging, as well as lower inflammatory biomarkers, than those treated with placebo at twelve-month follow-up [158]. Finally, the COCOMO-ACS aimed to assess the effect of colchicine post-MI on coronary plaque morphology OCT imaging in non-culprit segments of 64 patients. The study found that there were no differences in minimum FCT and maximum lipid arc throughout the imaged non-culprit segment, although cap rupture was less frequent. In a post-hoc analysis of patients followed up for 16 months, the increase of minimum FCT was higher in the colchicine than placebo group [159].

### 3.3. Preventive PCI

Beyond pharmacological strategies aimed at plaque stabilization and regression, several studies have explored, and others are currently investigating, the role of preventive PCI targeting vulnerable or high-risk plaques, as identified by invasive intracoronary imaging or noninvasive modalities.

The first pilot study in this area was the SECRITT trial [160], which randomized 23 patients with non-obstructive vulnerable lesions defined as TCFAs according to VH-IVUS and OCT criteria to PCI with a self-expanding nitinol stent or conservative management. In the PCI group, a substantial decrease in vessel wall strain and a notable increase in fibrous cap thickness were observed at six-month follow-up. Although not powered for clinical events, the SECRITT trial provided a proof-of-concept that plaque treatment with a stent may reduce lipid plaque content and increase cap stability. The PROSPECT-ABSORB was the first large trial comparing a preventive interventional strategy with conservative management of non-obstructing high-risk plaques [161]. Patients with acute MI undergoing PCI of the culprit lesion were screened with IVUS for non-obstructive lesions having plaque burden ≥65%. Eligible lesions were randomized to implantation of a coronary bioresorbable vascular scaffold (BVS) or conservative treatment. A total of 182 patients were included (93 in the BVS group and 89 in the conservative group). The primary endpoint—IVUS-derived minimal lumen area—was significantly larger in the PCI group as compared to controls (6.9 ± 2.6 mm^2^ vs. 3.0 ± 1.0 mm^2^; *p* < 0.0001). However, there were no significant differences in the rate of target lesion failure and MACEs at follow-up, although the study was not powered for clinical events. The first large trial of preventive PCI of vulnerable plaques powered for clinical events was recently published [9]. The PREVENT trial enrolled 1606 patients, mostly presenting with chronic coronary syndromes, being randomized 1:1 to medical treatment or PCI with BVS or drug-eluting stent of vulnerable coronary plaques defined as lesions with angiographic diameter stenosis >50% at angiography and FFR > 0.80, and at least 2 of the following four features: (1) MLA < 4.0 mm^2^ by IVUS or OCT; (2) IVUS plaque burden >70%; (3) LRP on near-infrared spectroscopy; (4) TCFA by OCT or radiofrequency-IVUS. The primary outcome was a composite of death from cardiac causes, target-vessel MI, ischemia-driven target-vessel revascularization, or hospitalization for unstable or progressive angina. At 2 years, the primary endpoint occurred in 3.4% of the conservative group and in 0.4% of the interventional group (HR 0.54, 95% CI: 0.33–0.87), suggesting a potential benefit of prophylactic PCI in selected high-risk plaques. However, the PREVENT trial has several notable limitations. Its open-label design may have introduced bias in the reporting of subjective outcomes. Longer duration of dual antiplatelet therapy in the PCI arm could have contributed to the lower event rates, acting as a confounding factor [162,163]. Additionally, the absence of systematic imaging follow-up limits insights into plaque evolution.

## 4. Future Directions

Many trials using different invasive and noninvasive imaging modalities for plaque assessment are ongoing and will clarify the role of preventive PCI in vulnerable and high-risk plaques. The INTERCLIMA study (NCT05027984) will assess the clinical effectiveness of an OCT-based strategy to guide revascularization in non-culprit intermediate coronary stenosis in patients with acute coronary syndrome (ACS) on the basis of the presence of morphological markers of plaque vulnerability, about a composite of cardiac death and target vessel spontaneous MI at 2 years and 5 years of follow-up. The COMBINE-INTERVENE study (NCT05333068) will match an OCT-FFR strategy, including OCT plaque assessment vs. FFR alone, in patients with multivessel coronary artery disease candidates for PCI about future MACEs at 24 months. The VULNERABLE trial (NCT05599061) will enroll ST-elevation MI patients with multivessel disease with FFR-negative intermediate lesions showing OCT features of vulnerability and will assess the outcomes related to a PCI with everolimus-eluting stents + optimal medical therapy strategy vs. optimal medical therapy alone about future target vessel failure at 4 years of follow-up. Finally, the FAVOR V trial (NCT05669222) will use noninvasive computerized angiography analysis for lesion functional assessment by quantitative flow ratio (μQFR) and plaque vulnerability evaluation through radial wall strain in ST-segment elevation MI patients with multivessel disease and will compare CCTA-guided PCI vs. coronary angiography +/- invasive functional assessment according to angiographic stenosis-guided PCI about future MACEs at 1.5 years of follow-up. The results of these trials will further clarify the role of preventive PCI in the presence of specific features of plaque vulnerability assessed by imaging about future outcomes.

## 5. Conclusions

Plaque microstructures identified through intracoronary OCT imaging have shown significant prognostic value in clinical studies. Within the comprehensive assessment of patients with coronary artery disease, such information may help characterize the individual atherosclerotic background and guide the implementation of more effective strategies for secondary prevention. Emerging evidence also suggests that preventive PCI could improve clinical outcomes in selected patients exhibiting high-risk plaque features. Future studies and clinical trials will be crucial to further refine the stratification of both “vulnerable plaques” and “vulnerable patients,” ultimately supporting the development of optimized therapeutic approaches aimed at reducing the clinical burden of atherosclerotic CV disease.

## Data Availability

No new data were created or analyzed in this study. Data sharing is not applicable to this article.

## Supplementary Materials

The following supporting information can be downloaded at: https://www.mdpi.com/article/10.3390/jcm14228132/s1. Table S1: Studies exploring the prognostic impact of OCT-detected microstructures in patients with CAD.
