# Supplementary material for: Prognostic Role and Therapeutic Implications of Intravascular Optical Coherence Tomography Detected Coronary Plaque Microstructures in Patients with Coronary Artery Disease
Source: J Clin Med. 2025 Nov 17;14(22):8132. doi: 10.3390/jcm14228132 (PMC12653084; doi:10.3390/jcm14228132)
Supplement: Supplementary file 1 [file jcm-14-08132-s001.zip › jcm-3959613-supplementary.pdf]

Table S1. Studies exploring the prognostic impact of OCT-detected microstructures in patients with CAD.

Name of the study	Study design	Study population	Type of lesions	Microstructures studied about clinical prognosis	Main prognostic endpoints	Follow-up time	Main prognostic results related to plaque microstructures
**CLIMA study (8) 2019**	Prospective, observational, multi-centre registry	1003 consecutive patients undergoing proximal LAD assessment by OCT in the context of clinically indicated coronary angiography	Untreated LAD proximal segment with angiographic stenosis ≤ 50%	Total plaque length, MLA, FCT, maximum lipid arc, MØI, CCs, layered (healed) plaque, CN, microvessels	Primary endpoint: composite of target-segment MI and/or cardiac death.	1 year	The presence of MLA <3.5 mm^2, FCT <75 μm, lipid arc circumferential extension >180° and MØI were all associated with increased risk of the primary endpoint.
**CLIMA study 5-year follow up (22) 2025**	Prospective, observational, multi-centre registry	1003 consecutive patients undergoing proximal LAD assessment by OCT in the context of clinically indicated coronary angiography	Untreated LAD proximal segment with angiographic stenosis ≤ 50%	Total plaque length, MLA, FCT, maximum lipid arc, MØI, CCs, layered (healed) plaque, CN, microvessels	Primary endpoint: composite of target-segment MI and/or cardiac death. Secondary endpoints: individual components of the primary composite endpoint, target	5 years	The presence of MLA <3.5 mm^2, FCT <75 μm, lipid arc circumferential extension >180° and MØI were all associated with increased risk of the primary endpoint. The presence of all 4 OCT-high risk criteria in the same patients were associated with each secondary

					vessel MI, any MI, and clinically driven TVR.		endpoint.
**Dai et al. (21) 2025**	Observational, single-centre study	1312 patients with acute MI with analyzable 3-vessel OCT imaging and scheduled clinical follow-up after discharge	Untreated NCL	TCFA, MØI, CCs, layered (healed) plaque, calcifications, microvessels	MACEs: cardiac death, non-fatal MI, and unplanned coronary revascularization, furtherly classified as culprit lesion-related MACEs or NCL-related MACEs	5 years	TCFA was related with NCL-related MACEs, independently from obstructive stenosis both at lesion- and patient-level. Calcification, but no other microstructures, was independently associated with NCL-MACEs at lesion-level
**COMBINE OCT-FFR (28) 2021**	Prospective, double-blind, international, natural history, multi-centre study	390 patients with DM undergoing coronary angiography with any clinical indication and having both FFR and OCT assessment of de-novo intermediate lesions (no culprit in MI)	FFR-negative intermediate lesions (no culprit lesions in MI patients)	TCFA	Primary endpoint: a composite of cardiac death, TV-MI, clinically-driven TLR, or hospitalization due to UA.	1.5 years	TCFA was a strong independent predictor of the primary endpoint.

**COMBINE OCT-FFR long term follow up (23) 2023**	Prospective, double blind, international, natural history, multi-centre study	390 patients with DM undergoing coronary angiography with any clinical indication and having both FFR and OCT assessment of de-novo intermediate lesions (no culprit in MI)	FFR negative intermediate lesions (no culprit lesions in MI patients)	TCFA, MLA, healed plaque	Primary endpoint: a composite of cardiac death, TV-MI, TLR, or hospitalization due to UA. Secondary endpoint: TV-MI, TLR, and UA treated as recurrent components of the endpoints	5 years	TCFA was an independent predictor of the primary endpoint. TCFA-positive patients had also higher risk of recurrent MACE. Healed plaques were not significantly associated with the primary outcome.
**Jiang et al. (25) 2023**	Retrospective, observational, single centre study	883 patients with acute MI with analyzable 3 main epicardial vessels by OCT imaging	Untreated NCL	TCFA, MLA, thrombus, MØI, CCs, healed plaque, non-culprit plaque rupture, microvessels, calcifications.	MACEs: cardiac death, non-fatal MI, and unplanned coronary revascularization	4 years	TCFA and MLA < 3.5 mm^2 were independent predictors of MACEs both at patient- and lesion-levels. Other plaque features did not independently predict the main outcome.
**Kubo et al (26) 2021**	Retrospective, observational, single centre study	1378 patients undergoing coronary angiography and OCT imaging	Untreated NCL	LRP, TCFA, MLA, fibrous plaque, calcific plaque, MØI, microvessels, CCs, FCT, lipid burden	Occurrence of ACS attributed to NCL imaged by OCT at baseline	6 years	No ACS occurred in fibrous and calcific plaque at follow up. Among lipid plaques, those with LRP and TCFA had more future ACS. Maximum lipid arc, minimum FCT and MLA were independent

							predictors of ACS, while CCs, MØI and microvessels were not.
**Yamaji et al. (27) 2024**	Prospective, multi-centre, observational study	176 patients who underwent PCI for target lesions and had at least 1 de novo non-target region with angiographicall y intermediate stenosis	Non-target regions	TCFA	All-cause death, cardiac death, nonfatal MI, and IDR	3 years	TCFA had a significantly higher risk of non-TV IDR, primarily due to revascularization in the imaged region. TCFA was not associated with other single endpoints.
**Xing et al. (38) 2017**	Prospective, observational, multi-centre registry (MGH OCT Registry)	1474 patients who had OCT imaging of the target vessel	Non-culprit lesions	LRP, TCFA, lipid burden, FCT, MLA, MØI, calcification, CCs, microvessels, thrombus, rupture	MACE: a composite of cardiac death, acute MI, and IDR, furtherly divided as culprit-lesion related MACEs or NCL-MACEs.	4 years	LRP was independently associated with increased risk of NCL-MACEs. LRP in patients with NCL-MACE had longer lipid lengths, wider maximal lipid arcs, and smaller MLA than LRPs in patients without NCL-MACEs. Other microstructures were not associated with the outcome.

**Biccirè et al. (sub analysis of CLIMA study) (39) 2023**	Prospective, observational, multi-centre registry	1003 consecutive patients undergoing proximal LAD assessment by OCT in the context of clinically indicated coronary angiography	Untreated proximal segment of LAD with angiographic stenosis ≤ 50%	OCT-derived LCBI 4mm	Primary endpoint: composite of cardiac death, MI, TVR. Secondary endpoint: composite of target-segment MI and/or cardiac death.	1 year	A max OCT-LCBI 4mm ≥400 predicted the primary and the secondary endpoint.
**Fabris et al. (sub analysis of COMBINE OCT FFR) (40) 2022**	Prospective, double blind, international, natural history, multi-centre study	390 patients with DM undergoing coronary angiography with any clinical indication and having both FFR and OCT assessment of de-novo intermediate lesions (no culprit in MI)	FFR-negative intermediate lesions (no culprit lesions in MI patients)	TCFA, ThCFA, LRP	Primary endpoint: a composite of cardiac death, TV-MI, TLR, or hospitalization due to UA.	1.5 years	Within LRP patients, TCFA patients had a much higher risk for primary endpoint compared with ThCFA and to non-LRP patients, whereas ThCFA patients had a risk similar to non-LRP. TCFA is the strongest independent predictor of primary endpoint.
**Kurihara et al. (47) 2020**	Prospective, observational, multi-centre registry (MGH OCT Registry)	265 patients with culprit vessel OCT imaging and 2 -year follow-up data	Culprit or non-culprit lesions in the culprit vessel	MLA, LRP, MØI, layered plaque	MACEs: cardiac death, ACS, or revascularization	2 years	Cumulative MACE was significantly higher in patients with layered plaque in the culprit vessel, which was primarily driven by

							TLR. No correlation between layered plaque and hard outcomes was found. No significant association between other plaque features and future revascularization is found.
**Yi et al. (48) 2025**	Retrospective, observational, single-centre study	222 ACS patients who underwent OCT at baseline and 1-year follow-up	Non-culprit lesions	New layered plaque	Cardiac death, non-culprit-related nonfatal MI, non-culprit-related clinically driven coronary revascularization and rehospitalization for unstable or progressive angina	6 years	NCL-MACEs was higher in patients with new layered pattern than in those without, mainly due to clinically driven coronary revascularization
**Usui et al. (49) 2020**	Retrospective, observational, single-centre study	538 patients who underwent percutaneous coronary intervention with evaluable non-culprit segments by OCT	Untreated non-culprit segments ≥5 mm long in target vessels	Layered plaque, MLA, TCFA	MACEs defined as a composite of cardiac death, MI, or IDR.	2.2 years	Layered plaque, MLA and TCFA were associated with MACEs. For layered plaques, events were driven by IDR.

**Yin et al. (53) 2022**	Single-arm, uncontrolled, prospective, single-centre proof-of-concept study (EROSION study)	137 ACS patients with culprit plaque erosion who underwent pre-intervention OCT imaging and received no stent implantation	Culprit plaque erosion	Newly formed healed plaque at the culprit site at 1-month	MACEs: composite of cardiac death, recurrent MI, IDR, stroke	1 year	No differences in MACEs between culprit plaque erosion with or without newly formed healed plaque at the culprit site at 1-month
**De Val et al. (sub analysis of COMBINE OCT FFR) (50) 2024**	Prospective, double blind, international, natural history, multi-centre study	388 patients with DM undergoing coronary angiography with any clinical indication and having both FFR and OCT assessment of de-novo intermediate lesions (no culprit in MI)	FFR-negative intermediate lesions (no culprit lesions in MI patients)	TCFA, MLA, healed plaque, microvessels, CCs, MØI,	Target LOCE, defined a composite of cardiac death, target vessel-related MI or clinically driven TLR	5 years	TCFA was an independent predictor of the primary endpoint. Healed plaques were associated with LOCE, primarily driven by clinically driven TLR. No correlation between layered plaque and single hard outcomes was found.
**Kimura et al. (51) 2023**	Retrospective, observational, single-centre study	417 consecutive lesions from SAP patients undergoing pre- and post-intervention OCT imaging	Culprit lesions in SAP patients undergoing PCI	Healed plaque	Cardiac death, recurrent or new-onset unstable angina, nonfatal MI, or ischemia-driven TLR.	Median-follow up 413 (IQR: 292-593) days	Healed plaques were associated with increased risk of MACEs, driven by ischemia-driven TLR. No correlation between healed plaque and single

							hard outcomes was found.
**Fracassi et al. (52) 2019**	Observational data from MGH OCT Registry and EROSION study	376 ACS patients who had undergone OCT imaging of the culprit lesion	Culprit lesions	Healed plaque	Cardiac death, acute MI, IDR, and rehospitalization	1 year	Rehospitalization rate was higher in patients with layered plaque. The incidence of death, acute MI and IDR were similar between the layered and non-layered plaques.
**Dai et al (54) 2020**	Prospective, single-centre study	417 patients with acute MI undergoing emergency PCI and OCT of all 3 major epicardial coronary arteries.	Culprit and non-culprit lesions	Healed plaque	MACEs: a composite of cardiac death, recurrent MI, IDR, or rehospitalization due to unstable or progressive angina.	1 year	Clinical outcomes were similar between patients with layered and non-layered plaques, both at culprit and non-culprit lesions
**Wang et al. (55) 2019**	Retrospective, observational, single-centre study	204 patients with pre-intervention OCT imaging of the culprit lesion	Culprit lesions	Multi-layered plaque	Death from any cause, MI, and TLR	1 year	No significant differences in 1-year outcomes were found between patients with and without multilayered plaques.
**Vergallo et al. (56) 2019**	Observational, single-centre cohort study with	105 patients undergoing OCT imaging during coronary	Non-culprit segments	LRP, TCFA, fibrous plaque, calcification, spotty	MACEs: composite of cardiac death, nonfatal MI, and	Median follow up 36.8 (IQR: 18.3-56-2)	The prevalence of healed plaques and fibrous plaques was the lowest, while LRP,

	prospective clinical follow up	angiography. Patients divided in 3 groups: (1) recurrent ACS, defined as history of at least 3 acute MI or at least 4 ACS with at least 1 acute MI; (2) patients with long-standing SAP defined as a minimum 3-year history of SAP; (3) patients with a single unheralded acute MI followed by a minimum 3-year period of clinical stability		calcification, microchannels, MØI	rehospitalization owing to UA	months	spotty calcification, MØI and TCFA was the highest in recurrent ACS group, the latter also having the lowest FCT and the highest lipid burden. TCFA and MØI were positively independently associated, while healed plaques were negatively independently associated with MACEs.
**Gatto et al. (sub analysis of CLIMA study) (66) 2022**	Prospective, observational, multi-centre registry	1003 consecutive patients undergoing proximal LAD assessment by OCT in the context of	Untreated LAD proximal segment with angiographic stenosis ≤ 50%	MLA, FCT, maximum lipid arc, MØI, MØI circumferential extension and measurement of the distance from intimal lumen	Primary endpoint: cardiac death, any MI, TVR	1 year	FCT, macrophage distance < 0.12 mm and macrophage arc > 67° were independent predictors of the main outcome

		clinically indicated coronary angiography		contour to macrophage string			
**OCT-FORMIDABLE study registry (67) 2018**	Retrospective, observational, multi-centre registry	209 patients with ACS undergoing OCT imaging of the culprit plaque	Culprit plaque	Plaque rupture, TCFA, MØI with necrotic core, LRP, fibrocalcific plaque, FCT, thrombus, MLA	MACEs defined as the composite of death from cardiac causes, non- fatal MI, clinically driven TVR.	Median follow up: 12.6 ± 14.5 months	Plaque rupture and necrotic core with MØI were independent predictors for MACEs
**Burgmaier et al. (68) 2020**	Prospective, observational, single-centre study	155 patients with CAD undergoing PCI and OCT imaging of the target lesion	Target lesion	TCFA, calcium, MØI	Composite endpoint: death from any cause, MI, revascularization	Median follow up: 5.4 (IQR: 4.4-5.8) years	MØI were more prevalent in patients with future events, while TCFA were not. Colocalization of MØI and calcium with a distance < 100 μm between macrophages and calcification was associated with future events.
**Fracassi et al. (69) 2019**	Prospectively-enrolling, single-centre registry	156 ACS patients undergoing pre-PCI OCT imaging of the culprit vessel	Culprit vessel	PR, lipid plaque, TCFA, MØI, calcifications, microchannels, FCT, lipid burden, MLA	Primary endpoint: recurrent ACS. Secondary endpoint: composite of cardiac death, recurrent ACS	3 years	PR, lipidic plaque and MØI were more frequent in recurrent ACS group. High-CRP, PR, MØI and multifocal atherosclerosis were independent predictors of the primary endpoint.

					and myocardial revascularization		High-CRP and PR were predictors of the secondary endpoint, whereas MØI were not.
**Montone et al. (70) (2020)**	Retrospective, observational, multicenter registry	153 ACS patients undergoing OCT imaging with evidence of plaque erosion at the culprit lesion	Culprit lesion	PE with MØI	MACEs: composite of cardiac death, recurrent MI and TVR	Median follow up: 2.5 (IQR: 2.03-2.58) years	PE with MØI were independent predictors of MACEs.
**Fujiyoshi et al. (79) 2019**	Retrospective, observational, single-centre study	340 patients undergoing OCT imaging and PCI of the culprit lesion	Culprit lesion	CCs, lipid plaque, TCFA, MØI microvessels, calcification, thrombus, lipid arc, FCT	MACEs: composite of cardiac death, non-fatal MI, IDR, furtherly classified in TLR, TVR and non-TVR.	1 year	MACEs were higher in patients with CCs at the culprit lesion (mainly driven by non-TVR). At univariate analysis, CCs, lipid plaque, MØI and TCFA were independent predictors of MACEs. At multivariate analysis, only TCFAs predicted MACEs and non-TVR.
**Nelles et al. (80) 2024**	Prospective, observational, multi-centre study	346 ACS patients undergoing OCT imaging of the culprit lesion	Culprit lesion	CCs, PR, LRP, CN, IFC, FCT, lipid burden, TCFA, MØI microvessels, calcification, MLA	MACE+: consisting of cardiac death, MI, TVR and re-hospitalization due to unstable or progressive angina	2 years	CCs, lipid index and PR were positively associated, while IFC was negatively associated, with MACE+ at univariate analysis, although only CCs independently

							predicted MACE+ at multivariate analysis.
**Usui et al. (81) 2021**	Retrospective, observational, single-centre study	566 patients with OCT imaged NCLs in the culprit vessel in patients who underwent OCT-guided PCI of the culprit lesion.	Untreated non-culprit lesion	LIA+CCs, LRP, MLA, TCFA	MACE includes cardiac death, MI, or IDR	Median follow up: 2.5 ± 0.7 years	LIA+CCs, TCFA and MLA were independent predictors of NCL-MACEs at follow up.
**Xu et al. (91) 2018**	Two-centre registry	535 patients with ischemic angina undergoing OCT imaging	Plaques treated or not with PCI	Microvessels	Intraprocedural no-reflow and periprocedural MI for lesions treated with PCI; revascularization for lesions which were not treated with PCI	3.2 years	Patients with intraplaque microvessels treated with PCI had higher rate of intraprocedural no-reflow and periprocedural MI. Patients with intraplaque microvessels which were not treated with PCI had increased risk of future clinically driven TVR than those without microvessels
**Nelles et al. (108) 2023**	Prospective, observational, multi-centre study	155 ACS patients undergoing OCT imaging of the culprit	Culprit lesion	Spotty calcium	MACE+: consisting of cardiac death, MI, clinicall-driven TVR and	1 year	The incidence of MACE+ was higher in patients with spotty calcium at the culprit lesion as compared to

		lesion			re-hospitalization due to unstable or progressive angina		those without spotty calcium.
**Prati et al. (post hoc analysis of CLIMA study) (109) 2020**	Prospective, observational, multi-centre registry	1003 consecutive patients undergoing proximal LAD assessment by OCT in the context of clinically indicated coronary angiography	Untreated LAD proximal segment with angiographic stenosis ≤ 50%	CN with and without disruption of the intimal fibrous layer	Primary endpoint: composite of target-segment MI and/or cardiac death. TVR was also assessed.	1 year	The primary endpoint occurred more frequently in patients with CN with disruption than CN without disruption and those without CN.<br>CN with disruption was an independent predictor of hard outcomes after correction for clinical characteristics and for four CLIMA features of vulnerability. The prevalence of TVR was similar in the two groups
**Yokomine et al. (110) 2025**	Retrospective, observational, single-centre study	122 patients with hemodialysis undergoing IVUS - (71%) or OCT/OFDI - (29%) guided PCI	Culprit lesion	CN	MACEs: composite of cardiac death, target lesion-related MI and TLR	1 year	MACEs were higher in hemodialysis patients with CN, than those with CN, mainly driven by cardiac death and TLR. CN was independently associated with MACEs also after adjusting for clinically relevant

							factors.
**Lei et al. (111) 2022**	Retrospective, observational, single-centre study	258 ACS patients with calcified culprit plaques imaged by OCT	Calcified culprit lesion	Eruptive CN, calcified protrusions and superficial calcific sheets. Calcium quantitative measures. Thrombus, MØI, layered plaque.	MACEs: a composite event of cardiac death, target-vessel MI, IDR.	2 years	Eruptive CN had more prevalence of future MACEs, mainly from target vessel MI and IDR than calcified protrusions and superficial calcific sheets. Eruptive CN was the only independent predictor of MACEs.
**Okamura et al. (112) 2022**	Retrospective, observational, single-centre study	49 CAD patients with end-stage renal disease on dialysis who underwent PCI using OCT	Target lesion	CN	MACE: composite of all-cause death, non-fatal MI, TVR and stroke	Median follow up: 826 days	MACEs were significantly higher in CN than in non-CN groups. No differences in all-cause death non-fatal MI and stroke were found, while a trend to worse outcomes in CN group was reported for TVR.
**Sugizaki et al. (113) 2024**	Observational study	372 patients with untreated calcified lesions undergoing OCT imaging at baseline and follow-up	Untreated calcified lesions	New CN at follow up	Cardiac death, MI, clinically driven revascularization	Median follow up: 1.5 years (IQR: 0.7-2.9 years)	Clinical events (revascularization and MI) were more frequent in lesions with new CN at follow up

**Vergallo et al. (121) 2016**	Prospective, observational, multi-centre registry (MGH OCT Registry)	261 patients with CAD undergoing 3-vessel OCT imaging	Culprit and non-culprit lesions	Non-culprit plaque rupture, TCFA	Clinical adverse events: death, non-fatal MI and non-TLR	1 year	Patients with non-culprit plaque rupture had higher rates of non-TLR. Clinical adverse events tended to be higher in non-culprit plaque rupture than those without non-culprit plaque rupture, and were higher in patients with non-culprit TCFA than those without TCFA
**Volleberg (122) 2025**	Registry combining prospective data from COMBINE OCT-FFR and PECTUS-obs	810 patients with FFR-negative non-culprit lesions undergoing OCT imaging	Non-culprit lesions	Lipid arc, FCT, thrombus, non-culprit plaque rupture	Native MACEs: composite of all-cause mortality, nonfatal MI, or unplanned revascularization<br><br>Target lesion failure: composite of cardiac death, target vessel MI, or TLR.	Median follow up: 761 (IQR: 731-1175) days	Lipid arc, minimum FCT < 65 μm and plaque rupture were associated with native MACEs and TLR. TCFA was associated with non-fatal MI, unplanned revascularization, target-vessel MI, TLR after correction of possible confounders.

In the table the words “layered” and “healed” have been used indiscriminately because layered phenotype denotes OCT appearance of healed plaques.

Abbreviations: ACS = acute coronary syndrome, CAD = coronary artery disease, CCs = cholesterol crystals, CN = calcified nodules, hs-CRP = high-sensitivity C-reactive protein, DM = diabetes mellitus, FCT = fibrous cap thickness, FFR = fractional flow reserve, IDR = ischemia-driven revascularization, IFC = intact fibrous cap, IQR = interquartile range, LAD = left anterior descending, LCBI = lipid core burden index, LIA = low-intensity area without attenuation, LOCE = lesion-oriented composite

endpoint , LRP = lipid-rich plaque, MACE = major adverse cardiovascular events, MLA = minimal lumen area, MI = myocardial infarction, MØI = macrophage infiltration, NCL = non-culprit lesion, OCT = optical coherence tomography, OFDI = optical frequency domain imaging, PE = plaque erosion, PCI = percutaneous coronary intervention, PR = plaque rupture, SAP = stable angina pectoris, TCFA = thin cap fibroatheroma, ThFCA = thick cap fibroatheroma, TLR = target lesion revasculariation, TV = target vessel, TVR = target vessel revascularization, UA = unstable angina.
